# The multiple organs insult and compensation mechanism in mice exposed to hypobaric hypoxia

**DOI:** 10.1007/s12192-020-01117-w

**Published:** 2020-05-19

**Authors:** Ning Li, Qiuyue Li, Jinrong Bai, Ke Chen, Hailing Yang, Wenxiang Wang, Fangfang Fan, Yi Zhang, Xianli Meng, Tingting Kuang, Gang Fan

**Affiliations:** 1 School of Ethnic Medicine, Chengdu University of Traditional Chinese Medicine, Chengdu, 611137 China; 2 Ethnic Medicine Academic Heritage Innovation Research Center, Chengdu University of Traditional Chinese Medicine, Chengdu, 611137 China; 3 College of Pharmacy, Chengdu University of Traditional Chinese Medicine, Chengdu, 611137 China; 4 Innovative Institute of Chinese Medicine and Pharmacy, Chengdu University of Traditional Chinese Medicine, Chengdu, 611137 China

**Keywords:** Hypoxia, Oxidative stress, HIF-1α, EPO, VEGF, Compensation mechanism

## Abstract

This study was first and systematically conducted to evaluate the hypoxia response of the brain, heart, lung, liver, and kidney of mice exposed to an animal hypobaric chamber. First, we examined the pathological damage of the above tissues by Hematoxylin & eosin (H&E) staining. Secondly, biochemical assays were used to detect oxidative stress indicators such as superoxide dismutase (SOD), malondialdehyde (MDA), reduced glutathione (GSH), and oxidized glutathione (GSSG). Finally, the hypoxia compensation mechanism of tissues was evaluated by expression levels of hypoxia-inducible factor 1 alpha (HIF-1α), erythropoietin (EPO), and vascular endothelial growth factor (VEGF). During the experiment, the mice lost weight gradually on the first 3 days, and then, the weight loss tended to remain stable, and feed consumption showed the inverse trend. H&E staining results showed that there were sparse and atrophic neurons and dissolved chromatin in the hypoxia group. And hyperemia occurred in the myocardium, lung, liver, and kidney. Meanwhile, hypoxia stimulated the enlargement of myocardial space, the infiltration of inflammatory cells in lung tissue, the swelling of epithelial cells in hepatic lobules and renal tubules, and the separation of basal cells. Moreover, hypoxia markedly inhibited the activity of SOD and GSH and exacerbated the levels of MDA and GSSG in the serum and five organs. In addition, hypoxia induced the expression of HIF-1α, EPO, and VEGF in five organs. These results suggest hypoxia leads to oxidative damage and compensation mechanism of the brain, heart, lung, liver, and kidney in varying degrees of mice.

## Introduction

Hypobaric hypoxia (HH) is the cardinal feature of the high-altitude environment. While the atmosphere is 21% oxygen at all altitudes, barometric pressure falls upon ascent, and with it the partial pressure of oxygen (PO_2_) (Murray 2016), and the deleterious effects of high altitude are primarily caused by the low inspired PO_2_ (West 2012). At 1500 m, *P*O_2_ is about 84% of the value at sea level, falling to 75% at 2500 m and 63% at 3500 m (WHO 2005). Traditionally, 2500 m has been used as the threshold for high-altitude illnesses. Approximately 140 million people live above 2500 m, but approximately 40 million others venture into high-altitude areas for work or leisure each year (Weil et al. 2007).

Previous evidences revealed that high altitude has stress detrimental influences on the functions of several cells due to free-radical damage sojourners, and mountaineers frequently experience different degrees of organ damage during high-mountain journeys. In recent years, a number of studies have revealed changes in the levels of molecules of organ damage caused by HH (Wang et al. 2019; Woods et al. 2017; Du et al. 2019). Previous studies have demonstrated that HH can induce an array of pathological reactions, including biochemical, molecular, and genomic alterations, most of which has focused on a certain tissue, such as the brain, heart, or lung. However, few studies have investigated the response of multiple tissues to hypoxia, lacking holistic research. HIF-1α is one of the most crucial signaling molecules which mediates the responses of mammalian cells to hypoxia by inducing the expression of adaptive gene products, such as EPO and VEGF. The increase of HIF-1α expression is related to hypoxic adaptation and the protection in the early stage of acute mountain sickness (AMS), but the prolongation of its downstream effector factor, VEGF, can induce excessive endothelial barrier dysfunction, increase vascular permeability, and eventually lead to high-altitude pulmonary edema (HAPE) and high-altitude cerebral edema (HACE). Oxidative stress refers to the imbalance between the production of reactive oxygen species and the ability of endogenous antioxidant system to remove these reactive oxygen species.

Considering the issues mentioned above, the purpose of this study is to systematically evaluate the histopathology, oxidative stress, HIF-1α, EPO, and VEGF of mice exposure to the HH. This study should provide an incentive for the exploration of the mechanisms and molecular-targeted therapeutic drugs for high-altitude sickness.

## Materials and methods

### Animals and ethics statement

A total of 22 male-specific pathogen-free BABL/c mice (7 weeks old, 20 ± 2 g, animal quality qualification certificate no. SCXK (Chuan) 2015-30) were obtained from Chengdu *Dashuo* Biological Technology l Co., Ltd (Chengdu, China). All animals were fed in a 12-h light/dark cycle at 23 ± 2 °C in 50–60% relative humidity. They were randomly divided into two groups of eleven mice each and were fed chow and water ad libitum. Animals were kept for 1 week for the purpose of acclimatization before experiment.

### Chemicals and reagents

Hematoxylin and eosin were provided by Thermo Fisher Scientific, Inc. (Waltham, MA, USA). ELISA kits of SOD, MDA, GSH, and GSSG were purchased by Nanjing Jiancheng Bioengineering Institute (Nanjing, China). HIF-1α and EPO were offered by Ellerite Biotechnology Co., Ltd (Wuhan, China). BCA protein assay kit and VEGF were procured from Lianke Biotechnology Co., Ltd (Hangzhou, China). All other chemicals used in this study were of analytical reagent grade.

### Animal grouping and experimental protocols

**Fig. 1:**
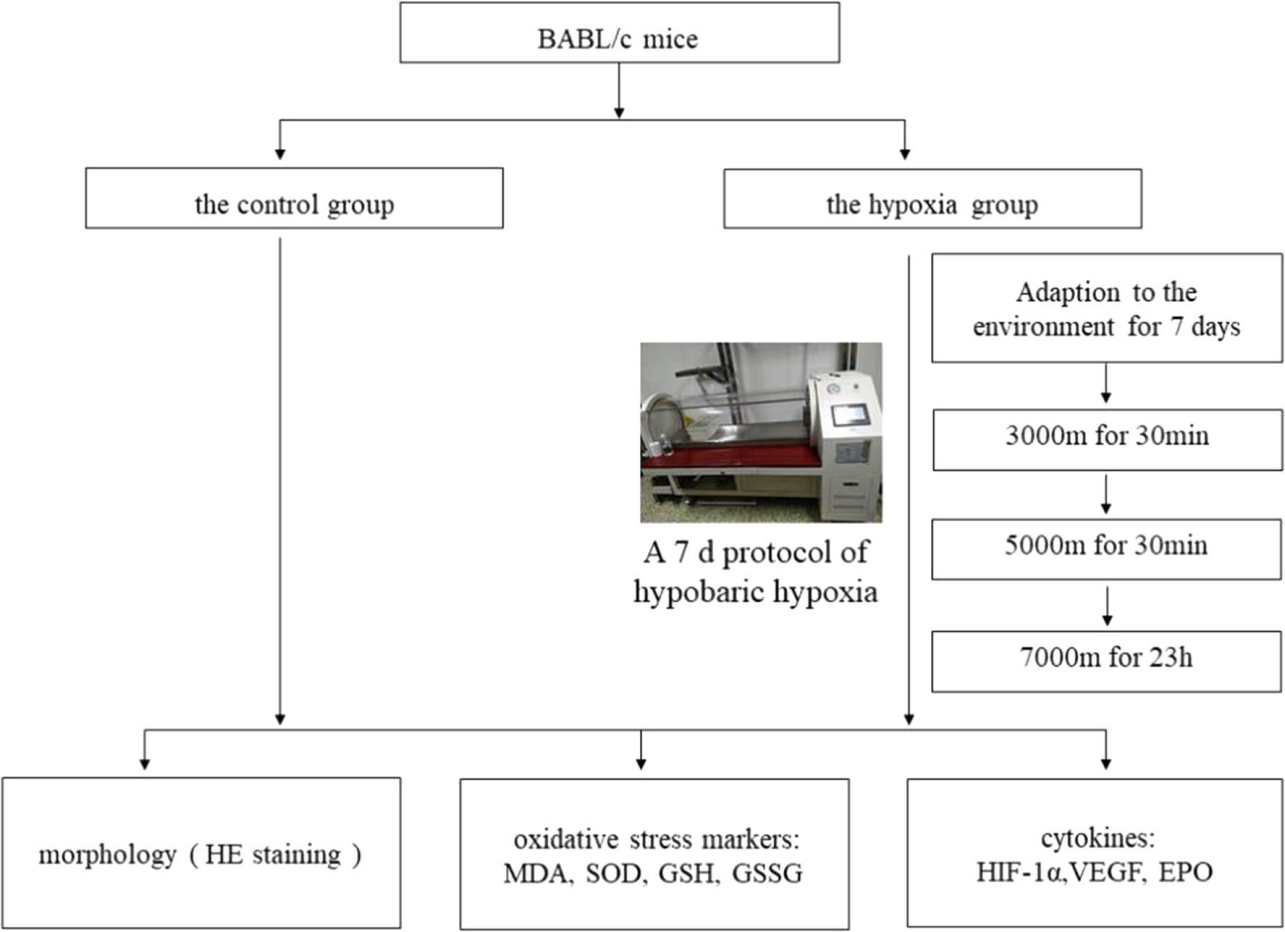
Experimental procedure of evaluation of HH-induced histopathology, oxidative stress, HIF-1α, EPO, and VEGF of mice exposure to HH

After adaption of the environment for 7 days, a total of 22 mice were divided randomly into 2 groups, including the control group and the hypoxia group. The hypoxia group was exposed to 7000 m of HH in an animal decompression chamber (Avic Guizhou Fenglei Aviation Armament Co., Ltd, China, FLYDWC50-II C). Anoxic environment was simulated by evacuating the air of the chamber using powerful vacuum pumps. The fresh air was allowed to circulate, and air flow was maintained inside the chamber at 0.9 L per minute to replenish consumed O_2_ and remove the produced CO_2_. The hypoxia group was subjected to the following protocols: 3000 and 4500 m above sea level at a speed of 5 m/s for 30 min and subsequent simulated 7000 m altitude for 23 h. The chamber was maintained at a temperature of 15–17 °C and a relative humidity of 55–60%. After hypobaric exposure for seven consecutive days, the elevation is immediately lowered at a speed of 5 m/s. The control group was subjected to similar conditions, except under normoxic conditions in a standard environment at an altitude of ~ 600 m. When the experiment was finished, blood samples were collected immediately using an orbital blood sampling after the hypobaric exposure for further detection. The brain, heart, lung, liver, and kidney were also collected for further ELISA assays. The experimental procedure is presented in Fig. 1.

### The body weight change ratio and feed consumption of mice

The body weights of mice and feed were weighed before entering and after leaving the animal decompression chamber every day, and the weight change ratio was calculated according to the following formula (1).

1 $$ \mathrm{Body}\ \mathrm{weight}\ \mathrm{change}\ \mathrm{ratio}\ \left(100\%\right)=\frac{\left(\mathrm{post}-\mathrm{exit}\ \mathrm{value}\right)-\left(\mathrm{pre}-\mathrm{entry}\ \mathrm{value}\right)}{\mathrm{pre}-\mathrm{entry}\ \mathrm{value}}\times 100\%. $$

### Biochemistry tests

Serum samples were prepared by leaving whole blood to stand at room temperature for coagulation and then centrifuged for 10 min at 4000 rpm. The brain, heart, lung, liver, and kidney homogenate 10% (*w/v*) was prepared in phosphate-buffered saline (PBS) and centrifuged at 4000 rpm for 10 min under 0 °C. All the samples were stored at − 80 °C before tested. The levels of SOD, MDA, GSH, and GSSG in the serum and tissue were determined according to the manufacturer’s instructions. HIF-1α (no. E-EL-M0687c), EPO (no. E-EL-M0027c), and VEGF (no. EK2832/2) in tissues were also detected using commercial ELISA kits according to the manufacturer’s instructions.

### H&E staining

For histological assessment, the whole tissue of the brain, heart, lung, liver, and kidney was respectively fixed in 4% paraformaldehyde with 7.2 pH at 25 °C for 24 h. The paraffin-embedded sections of the tissue were cut into 5 μm thickness by cryotome (RM2235, Germany), baked at 60 °C overnight, and dewaxed with xylene I and II for 20 min. Then, the sections were stained with hematoxylin for 30 min; washed with water for 20 min; stained with eosin for 5 min; dehydrated with 100%, 95%, 80%, and 70% ethanol for 5 min each; cleared in xylene; and mounted with neutral balsam. Finally, images were acquired using the Leica microscopic imaging system (DM1000, Germany) by CX22 microscope for positive stains (Olympus Corporation, Japan) to record the lesions in the brain, heart, lung, liver, and kidney.

### Statistical analysis

Data were presented as the mean ± standard deviation (SD). Statistical analysis was conducted by independent sample *T* test with SPSS 17.0 (SPSS, Inc., Chicago, IL, USA). Probability (*p* value) of less than 0.05 was considered statistically significant.

## Results

### The body weight change and feed consumption of mice

**Fig. 2:**
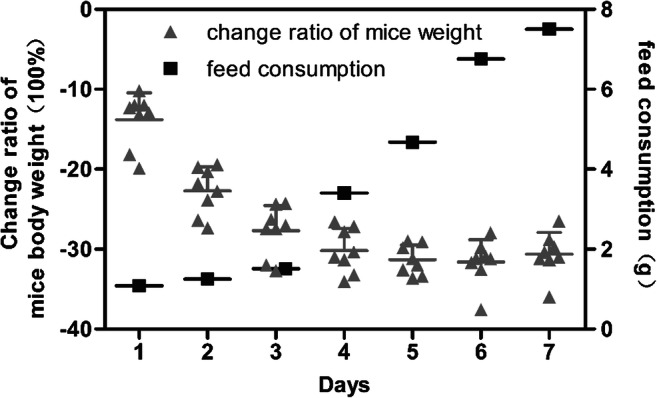
The body weight change ratio and feed consumption of mice exposure to HH. Each point represents an individual sample. Error bars indicate the mean ± SD (*n* = 8)

The mice body weight and feed were weighed before each entry into the animal decompression chamber, and the data analysis was shown in Fig. 2. The initial body weights of mice were 26.02 ± 2.25 g; after the first day of HH, body weights decreased by 13.82 %, after the fourth day decreased 27.69 %, and after the seventh day decreased 30.62%. And from the third day, the body weight change ratio of mice tended to remain stable, but the body weight was still remarkably lower than that before entering the chamber. Feed consumption in mice was lower in the first 3 days and increased gradually from the fourth day.

### HH induces morphological changes in the brain, heart, lung, liver, and kidney of mice

**Fig. 3:**
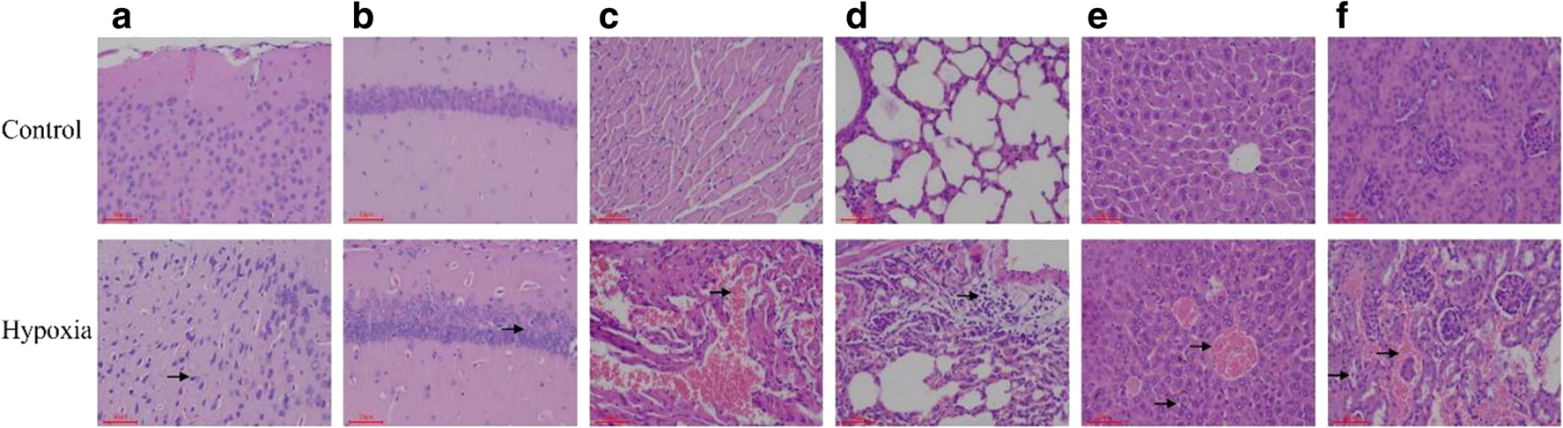
HE staining images of cortex (**a**), hippocampus (**b**), heart (**c**), lung (**d**), liver (**e**), and kidney (**f**) in HH-induced mice (× 200 magnification), compared with the control group. **a** Neurocyte shrinkage and chromatin dissolution. **b** The number of nerve cells decreased and their arrangement was sparse. **c** Focal myocardial hyperemia, interstitial capillary hyperemia, massive erythrocyte infiltration, and widening of myocardial space. **d** Local interstitial hyperplasia, hemorrhage, and extensive red blood cells appeared in alveolar cavity, accompanied by redundant inflammatory cells infiltration. **e** Central veins of hepatic lobules and hepatic sinus around the central vein, slight swelling of the cells in the marginal zone of hepatic lobule. **f** Capillary dilatation and congestion, swelling of renal tubular epithelial cells, exfoliation, and separation of basal cells

H&E staining of brain tissue revealed the neurocyte shrinkage and chromatin dissolution in the cortex; the decreased number of nerve cells, and the sparse arrangement were observed in the hippocampus in the hypoxia group compared with the control group (Fig. 3a, b). The result of myocardial tissue indicated focal myocardial hyperemia, interstitial capillary hyperemia, massive erythrocyte infiltration, and widening of myocardial space in the hypoxia group, compared with the control group (Fig. 3c). Local interstitial hyperplasia, hemorrhage, and large numbers of extensive red blood cells appeared in the alveolar cavity, accompanied by redundant inflammatory cell infiltration, were observed in the lung in the hypoxia group, compared with the control group (Fig. 3d). The liver showed congestion of central veins of hepatic lobules and hepatic sinus around the central vein along with slight swelling of the cells in the marginal zone of hepatic lobule (Fig. 3e). Capillary dilatation and congestion, swelling of renal tubular epithelial cells, exfoliation and separation of basal cells, and significant lesions of kidney tissue were observed in the hypoxia group, compared with the control group (Fig. 3f).

### HH induces decreased activity of SOD in serum and tissue

**Fig. 4:**
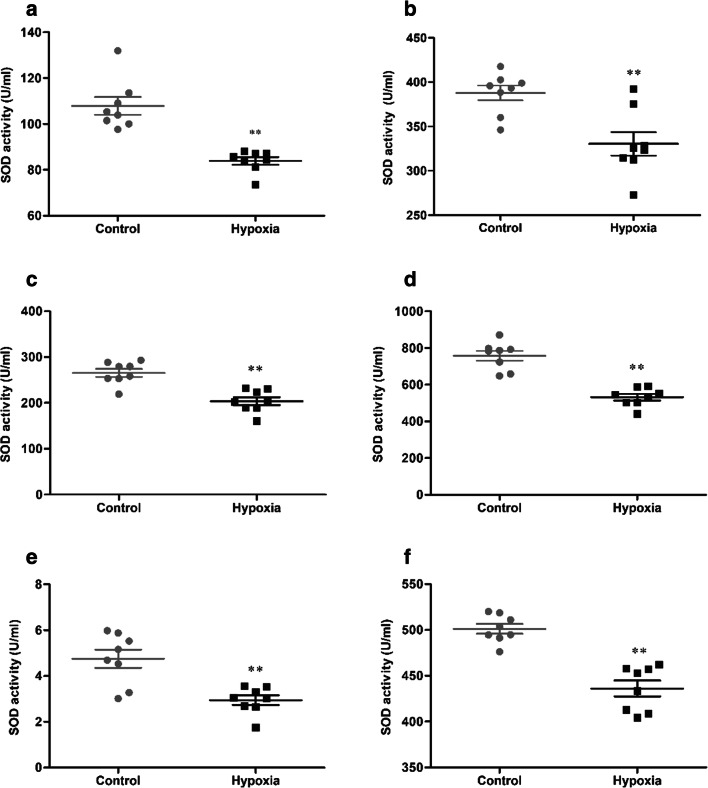
The activity of SOD in serum (**a**), brain (**b**), heart (**c**), lung (**d**), liver (**e**), and kidney (**f**) of mice exposure to HH. Each point represents an individual sample. Error bars indicate the mean ± SD (*n* = 8). ∗∗*p* < 0 01 *vs.* the control group

The activity of SOD in serum and tissue were determined, and the statistical results were presented in Fig. 4. After 7 days of HH, the activity of SOD (107.90 ± 10.97 vs 83.88 ± 4.75 U/mL, 387.93 ± 23.40 vs 330.54 ± 37.39 U/mL, 265.53 ± 24.36 vs 203.67 ± 24.43 U/mL, 757.83 ± 75.71 vs 531.77 ± 49.96 U/mL, 4.76 ± 1.12 vs 2.95 ± 0.59 U/mL, 501.26 ± 15.01 vs 436.16 ± 24.44 U/mL, *p* < 0.01, respectively, in the serum, brain, heart, lung, liver, and kidney) were significantly reduced in the hypoxia group, compared with the control group.

### HH induces the content of MDA in serum and tissue

**Fig. 5:**
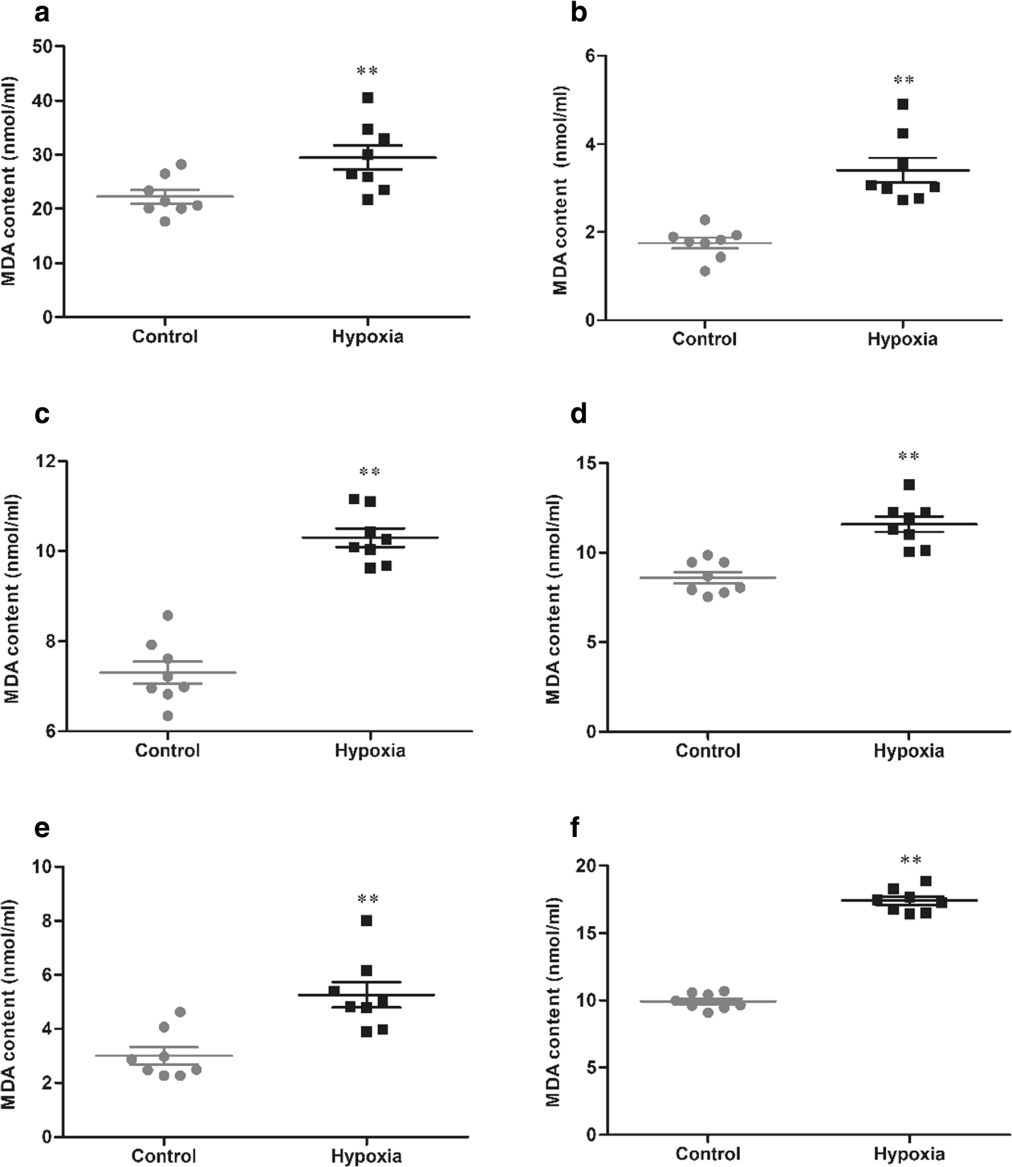
The content of MDA in serum (**a**), brain (**b**), heart (**c**), lung (**d**), liver (**e**), and kidney (**f**) of mice exposure to HH. Each point represents an individual sample. Error bars indicate the mean ± SD (*n* = 8). ∗∗*p* < 0 01 vs. the control group

The contents of MDA in serum and tissue were determined, and the statistical results were presented in Fig. 5. After 7 days of HH, the content of MDA (22.23 ± 3.57 vs 29.49 ± 6.33 nmol/mL, 1.75 ± 0.35 vs 3.41 ± 0.78 nmol/mL, 7.31 ± 0.71 vs 10.30 ± 0.58 nmol/mL, 8.58 ± 0.89 vs 11.58 ± 1.25 nmol/mL, 3.00 ± 0.88 vs 5.26 ± 1.33 nmol/mL, 9.93 ± 0.58 vs 17.40 ± 0.86 nmol/mL, *p* < 0.01, respectively, in serum, brain, heart, lung, liver, and kidney) were significantly increased in the hypoxia group, compared with the control group.

### HH induces decreased activity of GSH in serum and tissue

**Fig. 6:**
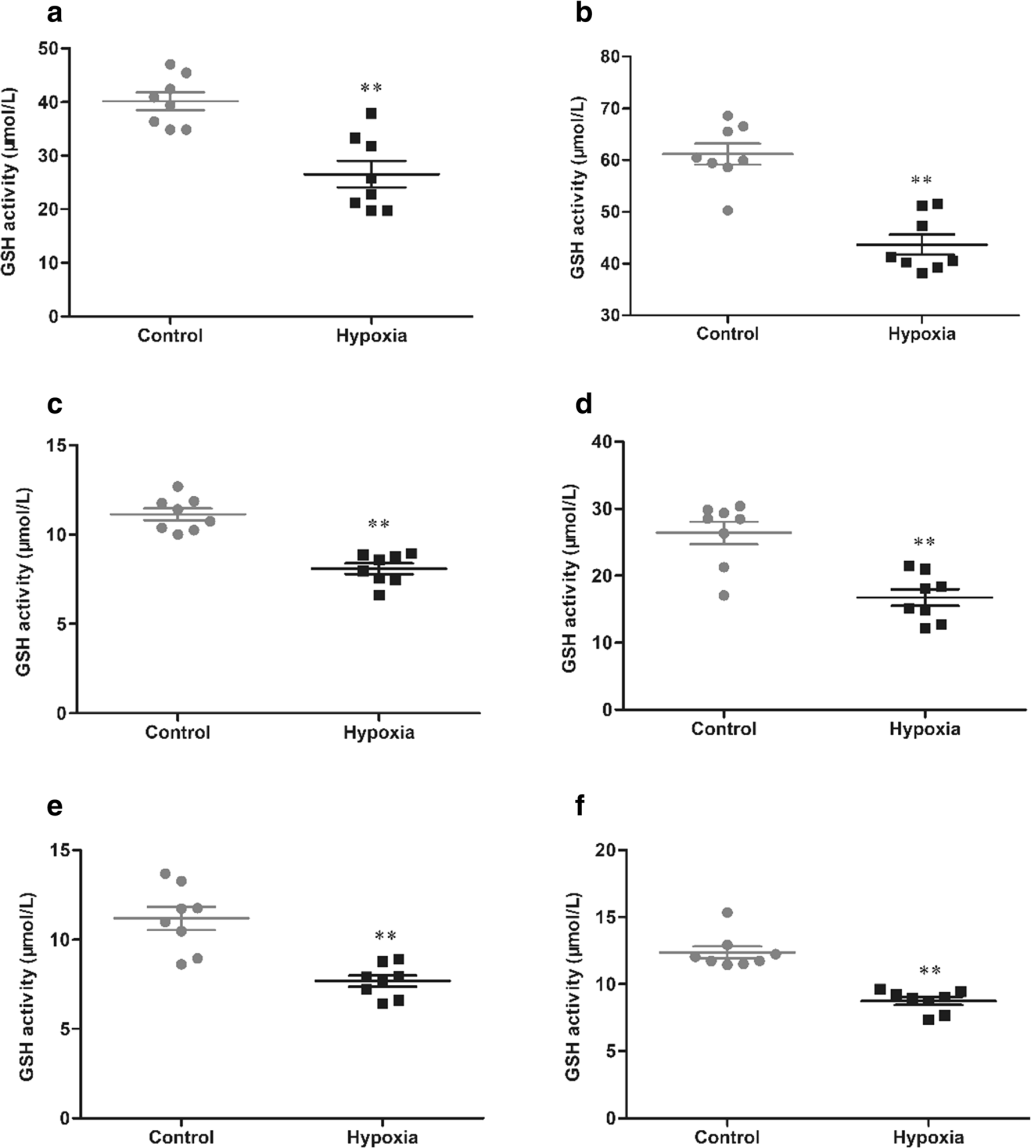
The activity of GSH in serum (**a**), brain (**b**), heart (**c**), lung (**d**), liver (**e**), and kidney (**f**) of mice exposure to HH. Each point represents an individual sample. Error bars indicate the mean ± SD (*n* = 8). ∗∗*p* < 0 01 vs. the control group

The activity of GSH in serum and tissue were determined, and the statistical results were presented in Fig. 6. After 7 days of HH, the activity of GSH (40.15 ± 4.65 vs 26.52 ± 6.97 μmol/L, 61.19 ± 5.78 vs 43.70 ± 5.46 μmol/L, 11.14 ± 0.93 vs 8.09 ± 0.84 μmol/L, 26.41 ± 4.77 vs 16.73 ± 3.55 μmol/L, 11.18 ± 1.83 vs 7.68 ± 0.91 μmol/L, 12.38 ± 1.29 vs 8.75 ± 0.82 μmol/L, *p* < 0.01, respectively, in serum, brain, heart, lung, liver, and kidney) were significantly reduced in the hypoxia group, compared with the control group.

### HH induces the content of GSSG in serum and tissue

**Fig. 7:**
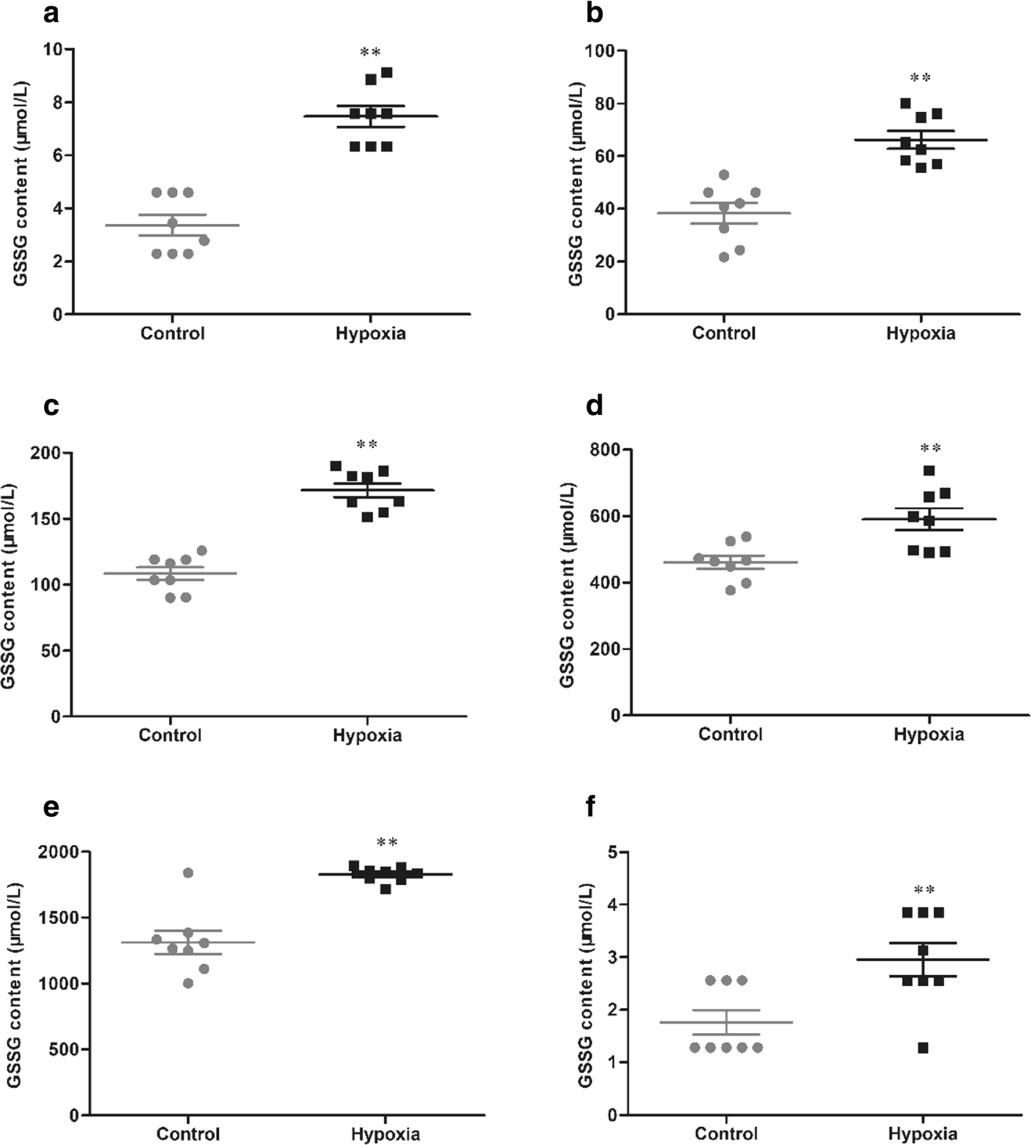
The content of GSSG in serum (**a**), brain (**b**), heart (**c**), lung (**d**), liver (**e**), and kidney (**f**) of mice exposure to HH. Each point represents an individual sample. Error bars indicate the mean ± SD (*n* = 8). ∗∗*p* < 0 01 vs. the control group

The content of GSSG in serum and tissue were determined, and the statistical results were presented in Fig. 7. After 7 days of HH, the content of GSSG (3.37 ± 1.09 vs 7.47 ± 1.11 μmol/L, 38.39 ± 11.08 vs 66.24 ± 9.52 μmol/L, 108.43 ± 13.62 vs 171.51 ± 15.04 μmol/L, 461.15 ± 55.39 vs 591.22 ± 92.4 μmol/L, 1312.65 ± 246.31 vs 1827.74 ± 56.77 μmol/L, 1.76 ± 0.66 vs 2.96 ± 0.90 μmol/L, *p* < 0.01, respectively, in the serum, brain, heart, lung, liver, and kidney) were significantly increased in the hypoxia group, compared with the control group.

### HH induces HIF-1α levels in tissue

**Fig. 8:**
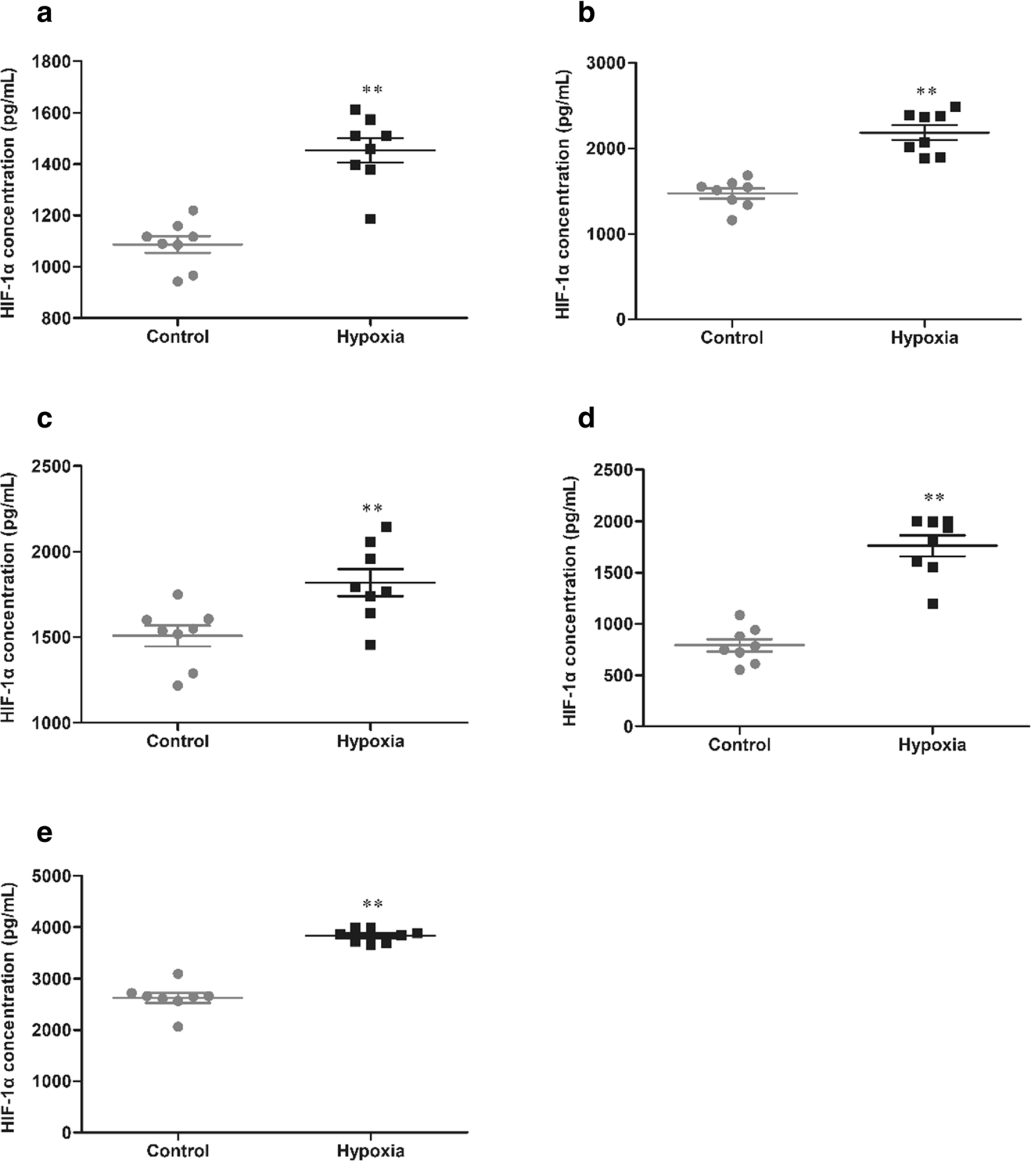
The concentration of HIF-1α in brain (**a**), heart (**b**), lung (**c**), liver (**d**), and kidney (**e**) of mice exposure to HH. Each point represents an individual sample. Error bars indicate the mean ± SD (*n* = 8). ∗∗*p* < 0 01 vs. the control group

The expression of HIF-1α in tissue was detected, and the statistical results were presented in Fig. 8. After 7 days of HH, the concentration of HIF-1α (1087.46 ± 92.75 vs 1454.42 ± 134.76 pg/mL, 1473.32 ± 166.06 vs 2187.38 ± 245.72 pg/mL, 1508.66 ± 173.43 vs 1819.79 ± 225.49 pg/mL, 787.70 ± 174.05 vs 1760.29 ± 289.48 pg/mL, 2624.27 ± 281.46 vs 3829.25 ± 124.73 pg/mL, *p* < 0.01, respectively, in the brain, heart, lung, liver and kidney) were significantly increased in the hypoxia group, compared with the control group.

### HH induces EPO levels in tissue

**Fig. 9:**
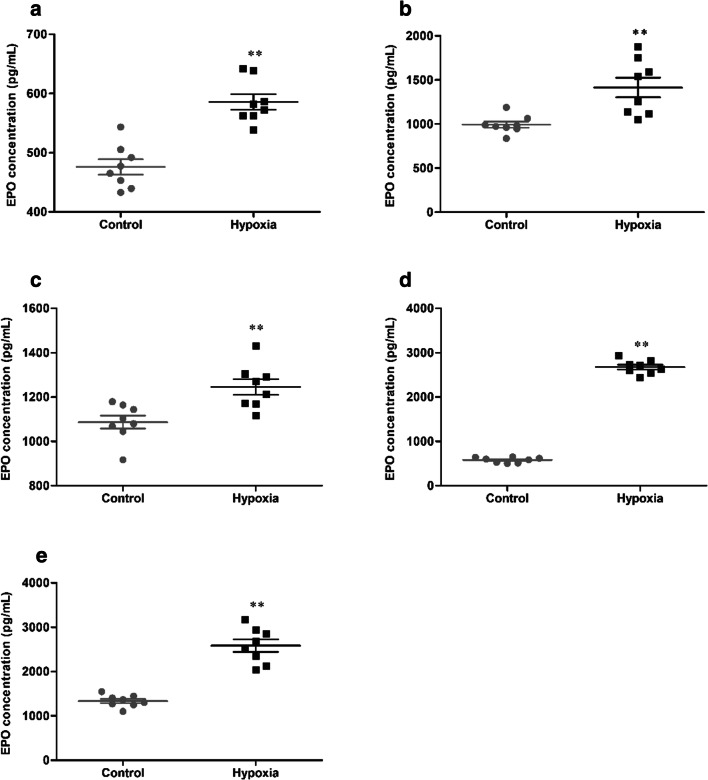
The concentration of EPO in brain (**a**), heart (**b**), lung (**c**), liver (**d**), and kidney (**e**) of mice exposure to HH. Each point represents an individual sample. Error bars indicate the mean ± SD (*n* = 8). ∗∗*p* < 0 01 vs. the control group

The expression of EPO in tissue was detected, and the statistical results were presented in Fig. 9. After 7 days of HH, the concentration of EPO (476.11 ± 36.82 vs 585.76 ± 36.75 pg/mL, 992.64 ± 101.26 vs 1414.56 ± 315.85 pg/mL, 1087.30 ± 83.61 vs 1245.81 ± 99.47 pg/mL, 580.18 ± 59.00 vs 2675.71 ± 156.92 pg/mL, 1336.38 ± 136.21 vs 2584.79 ± 399.42 pg/mL, *p* < 0.01, respectively, in the brain, heart, lung, liver, and kidney) were significantly increased in the hypoxia group, compared with the control group.

### HH induces VEGF levels in tissue

**Fig. 10:**
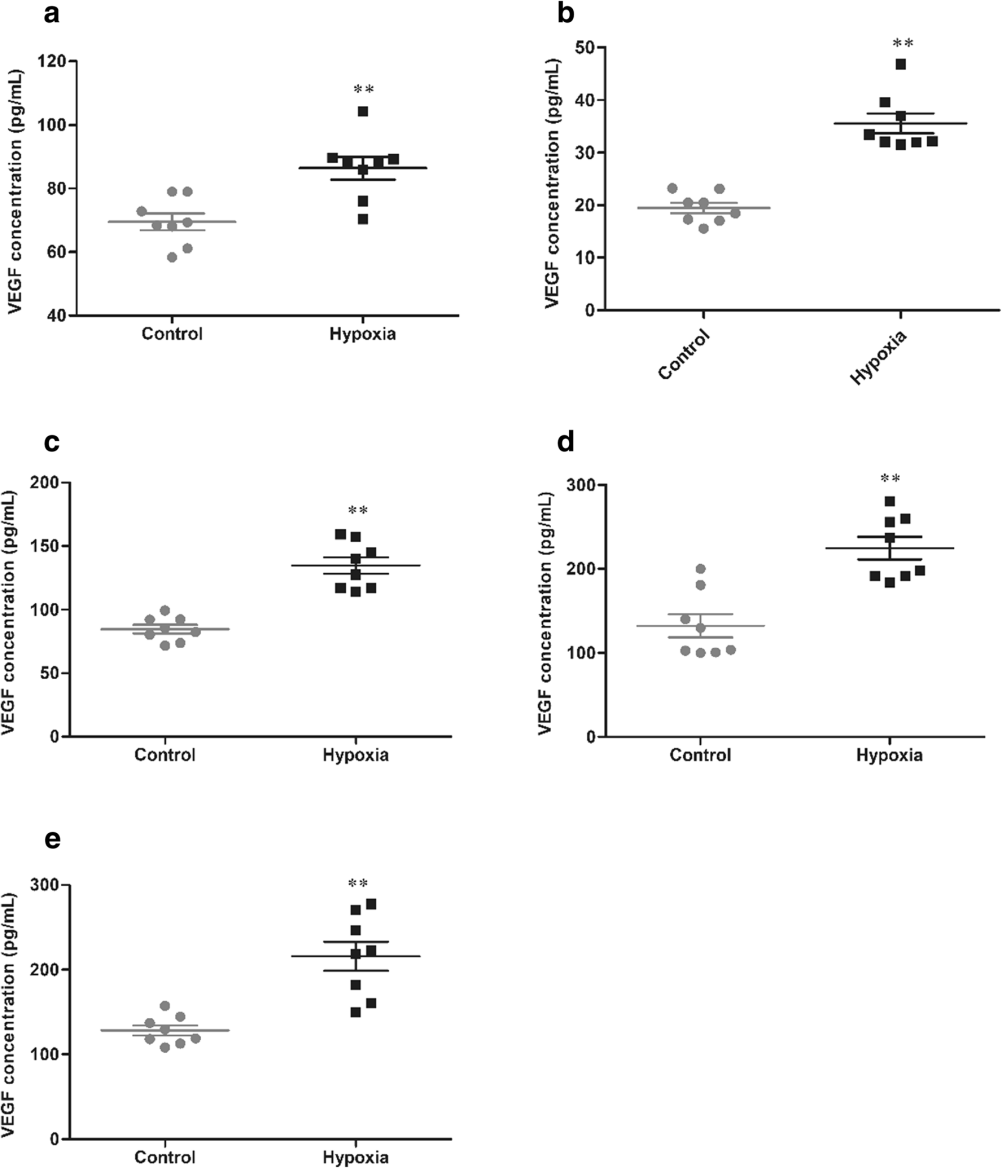
The concentration of VEGF in brain (**a**), heart (**b**), lung (**c**), liver (**d**), and kidney (**e**) of mice exposure to HH. Each point represents an individual sample. Error bars indicate the mean ± SD (*n* = 8). ∗∗*p* < 0 01 vs. the control group

The expression of VEGF in tissue was detected, and the statistical results were presented in Fig. 10. After 7 days of HH, the concentration of VEGF (69.50 ± 7.45 vs 86.42 ± 10.07 pg/mL, 19.43 ± 2.84 vs 35.57 ± 5.36 pg/mL, 84.71 ± 9.61 vs 134.68 ± 18.21 pg/mL, 132.40 ± 39.12 vs 224.80 ± 37.93 pg/mL, 128.30 ± 16.98 vs 216.07 ± 48.29 pg/mL, *p* < 0.01, respectively, in the brain, heart, lung, liver, and kidney) were significantly increased in the hypoxia group, compared with the control group.

## Discussion

Oxygen is vital to the survival of mammalian cells and tissues. HH, the most common cause of which is dwelling at high altitude, is critical in the regulation of the microenvironment of every cell. Understanding the molecular regulation of HH and how cells and organisms respond to it is a major area of research focus.

Hypoxia has a wide range of non-specific effects on different organs of the body, and the extent and consequences of the effects depend on the functional metabolism of the organ. Previous studies on HH mainly focused on its effects on the brain, heart, and lung, rarely involving the liver and kidney.

HH leads to hypoxemia as well as tissue hypoxia (West 2017). And persons who are not acclimatized and ascend rapidly to elevations above 2500 m are at risk for any of several debilitating and potentially lethal illnesses including AMS, HACE, and HAPE that occur within the first days after arrival at high altitudes, leading to considerable morbidity and mortality, if not diagnosed or treated in a timely way (West 2014; Luks et al. 2014; Bärtsch and Swenson 2013). Studies have reported that AMS occurs in approximately 10 to 25% of unacclimatized persons who ascend to 2500 m, and 50 to 85% at 4500 to 5500 m, and may be incapacitating (Tsai et al. 2019; Maggiorini et al. 1990; Sánchez-Mascuñano et al. 2017). The prevalence of HACE is estimated to be 0.5 to 1.0% among persons at 4000 to 5000 m (Zhou et al. 2017). What is more serious is that without appropriate treatment, coma may evolve rapidly, followed by death from hernia cerebri within 24 hours (Li et al. 2018). The incidence of HAPE among persons is 6% and 15%, respectively, when reached at 4500 m and 5500 m within 1 or 2 days, with the estimated mortality 50% (West 2014).

In the current study, within 7 days of hypoxia, the trend of weight loss in mice was contrary to that of feed consumption, which may be related to the regulation of high-altitude acclimatization and basal metabolic rate of mice under hypoxia. H&E staining results showed that hypoxia could lead to different degrees of pathological changes in tissues. Hypoxia led to neurocyte shrinkage and chromatin dissolution in the cortex and a decreased number and sparse arrangement of nerve cells in the hippocampus. In myocardial tissue, hypoxia caused interstitial capillary hyperemia, massive erythrocyte infiltration, and widening of myocardial space. Lung hypoxia engendered local interstitial hyperplasia and hemorrhage. Large numbers of red blood cells appeared in the alveolar cavity, accompanied by redundant inflammatory cells infiltration. The liver showed hypoxia caused congestion of central veins of hepatic lobules and hepatic sinus around the central vein, slight swelling of the cells in the marginal zone of the hepatic lobule. And hypoxia stimulated capillary dilatation and congestion, swelling of renal tubular epithelial cells along with exfoliation, and separation of basal cells in the kidney.

Oxidative stress was described as an disequilibrium between prooxidants and antioxidants in biological systems, referring to the enhanced production of reactive oxygen species (ROS) and/or depletion of the antioxidant defense system (Chaudhary et al. 2019; Singh et al. 2019; Zeng et al. 2013). Once oxidative stress increases, various cytokines and enzymes are differentially activated to inhibit further damage and maintain homeostasis in organisms. SOD are the key players of cellular defense against superoxide ions (O2-.) converting them to H2O2 for further action by catalases, converting the released H2O2 to H2O and O2 (Sharma et al. 2018). The result demonstrated that HH markedly inhibited the activity of SOD in serum and five organs. Overproduction of intracellular lipid peroxidation is the major manifestation of excessive ROS, leading to the peroxidation of the mitochondria and destruction of the integrity of the cell structure, and MDA is the prominent lipid peroxidation products (Kwon et al. 2015; Mi et al. 2019). The result showed that HH markedly exacerbated the content of MDA in serum and five organs. GSH is a primary antioxidant molecule which belongs to the endogenous defense against ROS, and its role is critical for the cellular redox environment (Small et al. 2012). It is the most abundant nonprotein thiol that counteracts oxidative stress (Lu 2013), playing a vital role in scavenging reactive oxygen intermediates and converting them into fatty acids and water as GSH is oxidized to GSSG (El-Mihi et al. 2017). The result demonstrated that HH markedly inhibited the activity of GSH and exacerbated the content of GSSG in serum and five organs, which is harmful to maintaining the normal antioxidant defense system.

HIF-1 is the master regulator of oxygen homeostasis triggering metabolic adaptations to hypoxia, having an oxygen-sensitive α subunit and a constitutively expressed β subunit (Haase 2006; Hou et al. 2019). HIF-1α is one of the most crucial signaling molecules in tissue directly involved in metabolic adaptations to hypoxia, as it regulates many genes that are important in promoting cell survival such as EPO and VEGF (Klatte et al. 2007; Stoyanoff et al. 2016; Anderson et al. 2009). Induction of HIF-1α is an early cellular response to changes in oxygen homeostasis in the brain, and HIF-1α activation promotes cell survival in hypoxic tissues (Sharp et al. 2001). HIF-1 is not only responsible for regulating hypoxic adaptation at the body level, but also for mediating the rate of glycolysis at the cellular level (Firth et al. 1994). EPO is the chief regulator of red blood cell production in mammals, and its generation is highly responsive to tissue hypoxia, through HIF-1α (Nekoui and Blaise 2017; Cantarelli et al. 2019; Tian et al. 2019). In mammals, red blood cells are needed as oxygen carriers and are transported throughout the whole body. If oxygen is deficient, EPO secreted from the kidney will stimulate bone marrow to produce new red blood cells, thus improving oxygen transport capacity (Miyake et al. 1977). VEGF is an endothelial-specific mitogen that increases peripheral oxygen delivery by stimulating angiogenesis, involving endothelial cell migration, proliferation, and differentiation, as well as extracellular matrix proteolysis (Lemus-Varela et al. 2010). Our present findings revealed that HH induced the expression of HIF-1α, EPO, and VEGF in the brain, heart, lung, liver, and kidney, indicating that the body resisted HH injury by increasing erythropoiesis and angiogenesis to raise the transport of oxygen and nutrients, and these changes occur as a compensation mechanism of the body to hypoxia.

In this study, we firstly and systematically conducted an evaluation of the hypoxia response of the brain, heart, lung, liver, and kidney of mice exposure in an animal hypobaric chamber. Our results revealed that HH resulted in continuous weight loss and increased feed consumption in mice. Simultaneously, HH led to different degrees of pathological changes in different organs. Moreover, HH can markedly affect oxidative stress index and hypoxia-sensitive indices in different organs. In summary, hypoxia leads to oxidative damage and compensation mechanism of the brain, heart, lung, liver, and kidney in varying degrees.
